# PEGylated silica-enzyme nanoconjugates: a new frontier in large scale separation of α-amylase

**DOI:** 10.1038/srep18221

**Published:** 2015-12-14

**Authors:** Seyed Mohsen Dehnavi, Gholamreza Pazuki, Manouchehr Vossoughi

**Affiliations:** 1 Institute for Nanoscience and Nanotechnology, Sharif University of Technology, Tehran, Iran; 2 Department of Chemical Engineering, Amirkabir University of Technology (Tehran Polytechnic), Tehran, Iran; 3 Department of Chemical and Petroleum Engineering, Sharif University of Technology, Tehran, Iran

## Abstract

High resolution is nearly lost at the expense of throughput in most conventional bioseparation methods. Nanoparticles, due to their high surface to volume ratio, are attractiveenzyme carriers, which can boost the performance of extraction manifold. Here, wereport design and application ofa method highly capable of improving the partitioning of α-amylase in aqueous two-phase system of polymer and salt. Silica nanoparticle introduced to the system acts as a bridge that connects the enzyme and polymer. Theconjugated nanoparticles form the major part of the upper phase and thus significantly enhance the protein recovery. A thorough investigation was performed on the structure of the nanoconjugatesas well as analyzing the conformational structure of the enzyme after conjugationto explore anypossible denaturation.

Proteinsplay a crucial role in a variety of activities that sustain the cellular life cycle such as enzymatic catalysis, transport of nutritious substrates, construction of tissues and cellular scaffold, immunization, cell movement, replication of DNA, cellular signaling and transduction, energy supply and storage^1^,^2^,^3^,^4^. Therefore, proteinscould properly be called “the quintessence of life”. In a broad sense, proteins are surface-active macro biomolecules with extreme affinity to lodge in liquid-liquid andliquid-solid interfaces. Through different types of interactions (e.g. electrostatic, hydrophobic, H-bonds or covalent bonds),amphiphilic proteins interact with countless organic and inorganic substrates.Thus, separation and purification of proteins is of high importance and relevance.

For biomedical application, confronting immunologic diseases and development of biocompatible devices have long been a challenge. Complement activation and resultant rejection of transplants, overactive immune system, and thrombotic complications of cardiovascular implants are few examples that involve the activity of protein at interfaces^5^,^6^. Proteins are also very important for food and pharmaceutical industries.Protein industry has established a huge market value and it holds promise that it expands. Recombinant monoclonal antibodies (mAb), cytokines, chemokines, interferons, and growth hormones are among such examples for biomedical applications of proteins, Regarding food applications,proteins impart a wide spectrum of functional properties including gelation, clarity, foaming, emulsion stability, water-holding, which directly affect the appearance, texture and taste of the food^7^.

The emergence of nanoparticle-biomolecule hybrid systems hadan immense impact on various disciplines of science and technology, delegating brand new missions to adjuvant therapeutics, imaging, sensing, delivery, diagnosis, bioelectronics, separation and study of protein structures^8^,^9^,^10^,^11^,^12^. Examples include attempts made for selection of the cancerous cells concomitant with non-invasive treatments (e.g. light-activated immunoconjugate targeting, magnetite nanoparticle careers designed for hyperthermic treatments, super magnetic iron oxide drug careers, phototoxicity inflicted by reactive oxygen), self-assembly fabrication of nano-plugged biomolecules specifically functionalized to utilize size-tunable photoluminescence characteristics of semiconductor quantum dots for the purpose of detecting chemicals, conjugation of magnetic nanoparticles with an antibody to serve as a contrast magnetic resonance imaging (MRI) agent and enzymatic nano-wiring of fuel cell elements.The reader is referred to these references for further knowledge^13^,^14^,^15^,^16^,^17^.

Silica nanoparticles with hierarchical structures inherit remarkable features, which hasattracted attention from different disciplines. Silica is capable of shaping an adjustableaggregate morphology with a size ranging from a few to hundreds nanometers. This feature along with high surface area confers a flexible surface chemistry upon silica nanoparticles suiting them to biocompatibility-demanding applications. High capacity for surface functionalization facilitatesthe attachment of anchoring mediates and subsequent grafting ofdesired acidic, basic, hydrophilic and hydrophobicfunctional groups,thus rendering an engineered surface with selective affinity toward diverse bio-compounds and amenable to biocompatibility measures^18^,^19^,^20^,^21^,^22^.

According to a recent study, with an average annual growth rate of 6.8% over five years enzymes are going to establish a $6.3 billion market by 2020. Environmental regulations, propensity of industries for cost reduction and technological advances in biotechnology are amongst incentives that has given a considerable momentum to the growth of enzyme industry^23^. As it regards the technological advances, high-yield separation methods mainly contribute to the final cost of biological products.

Through evolution, enzymes have adapted to catalyze numerous metabolic pathways resultant in cell sustainability. Inspired by the mechanisms enzymes act on their substrate, it has been made possible to adopt natural and engineered enzymes for manifold industrial purposes. Though acting on many substrates, enzymes are very delicate to an extent even a slight change in their environment can lead to conformational distortion and consequent denaturation. Besides, enzymes are fermented in a multi-component aqueous broth. In this regard, it urges the development of isolation methods, which not only preserve their native state but also allow for analytical studies of amino acid sequences fundamentally causative of their surface affinity and physicochemical properties.

Aqueous two-phase systems provide a mild environment for separation of enzymes compatible with their nature. The technique is based onrelative strength of interactions between solute and solvent in different phases^24^,^25^. Dependency of such interactions on the size and structure of constituents, pH, ionic strength and temperature offers a flexible approach with high biocompatibility. A large amount of work has been carried out on the partitioning of different biomolecules. These systems mostly contain water and polymer, which split phases by addition of salts at low concentrations. Thoughexhibiting fair partitioning of solute when an appropriate combination of components selected, efficiency of the system hinges on exact recognition of intra-molecular interactions, whichdue to the complexity of the biomolecule containingsystems, is open to ambiguity. Thus, the choice of optimum system depends mainly on conjecture^26^,^27^,^28^,^29^,^30^,^31^,^32^,^33^,^34^.

Using this scenario, we developed a nanoparticle-plugged partitioning of α-amylase resulting in the enhancement of enzyme recovery. Our approach is based on conventional aqueous two-phase system concept. By introduction of nanosilica particles into the system and exact control of pH, we trigger the germination of PEGylated enzyme carriers. We used the state-of-the-art nanoconjugation technique to engineer enzyme-nanosilica-polymer conjugate, whichsafely delivers the enzyme to polymer phase in large amounts, thereby reducing the dependency on trial-and-error optimizations.

## Results and Discussion

### Enzyme stabilization

**Figure 1:**
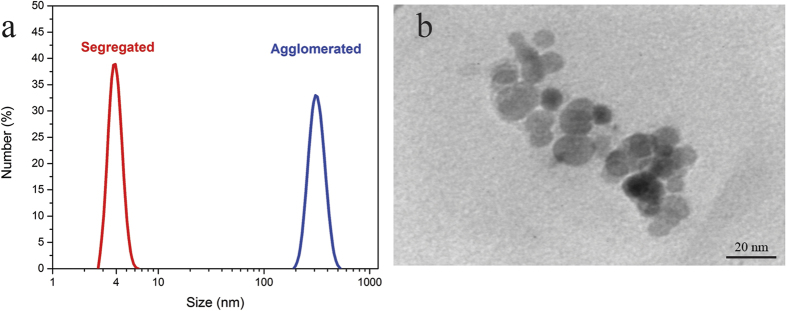
(**a**) DLS analysis of α-amylase in aqueous solution; segregated enzyme (red), agglomerated enzyme (blue). (**b**) TEM image of stabilized α-amylase.

To a notable degree, agglomerationresults in inactivation of α-amylase. There is a cysteine residue (C84) on the catalytic domain of α-amylase with a source of *bacillus subtilis* responsible for probable formation of agglomerates^35^. It has been observed that rarely the enzyme undergoes agglomeration in a short time span, despite harsh physical and chemical conditions such as high temperature, shear stress and pHvariations imposed^28^. Energy barriers with repulsive van der Waals and electrostatic origin hinder the agglomeration of enzyme particles. Moreover, if one provides the system with ample amounts of energy to overcome the barriers, the flexible molecular structure of the enzyme will find an appropriate sterical position to undergo ligation of sulfides on the cysteine residues which wouldonly be a matter of time^27^,^35^. However, as we prepared the aqueous solution of the enzyme, colloidal agglomerates with the size of 35 nm were observed by DLS (Fig. 1a) after an overnight rest. It suggests that stirring supplied the solution with enough energy for effective collision of enzyme particles and the relaxation time allowed the particles to properly orient. That being observed, with an eye toward the points that we need sonication to finely distribute nanoparticles in the later stages of separation and the time being the inseparable feature of ATPS process, it is necessary to segregate the enzyme agglomerates and take on efficient stabilization methods to assure that no agglomeration and consequent inactivation takes place during the whole process of separation.

Generally, there arethree mechanisms to stabilize colloidal solutions: electrostatic, steric and depletion stabilizations^36^,^37^. α-Amylase has an isoelectric point of 6.5 pH at which the enzyme carries no net charge and hydrophobic interactions are at the highest. Thusthere is a good chancefor formation of agglomerates at this pH. Salt additives can alterpH of aqueous solutions. Magnesium sulfate would be an amiable choicedue to two reasons: first, when added to the enzyme containing solution by amount of 2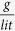, it lowers the pH from 7 to 6. Consequently, the enzymatic associations get positively charged, and then breakinto smaller agglomerates with an effective diameter of 4 nm (Fig. 1a). Second, MgSO_4_ slightly affects the salt bridges on the catalytic domain of the enzyme, which results inthe activity to be preserved. Upon phase split, the enzyme attends the upper phase. Since the upper phase is depleted of the salt, the charge distribution over the enzyme particles may subside. As a consequence, the enzyme becomes vulnerable to agglomeration. However, by proper choice of a polymer, the possibility of agglomeration decreases to a great extent.Poly ethylene glycol (PEG), the main constituent of the upper phase, circumvents the enzyme and kinetically stabilizes it by depletion of the polymer adjacent to colloidal particles^10^. Figure 1b demonstrates the TEM image of the stabilized enzyme. As seen, the coiled PEG chains situated between enzyme particles obstruct the agglomeration.

### Formation and characterization of nanoconjugates

**Figure 2:**
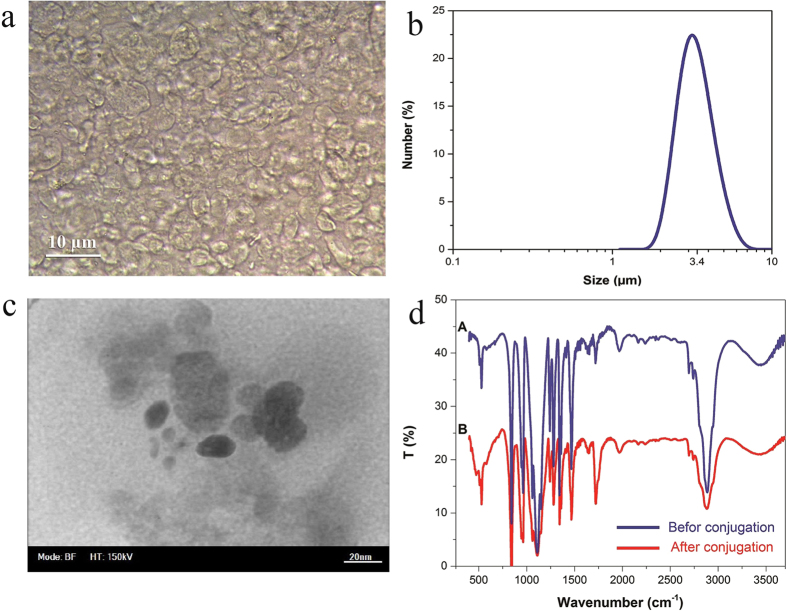
Characterization of synthesized clusters. (**a**) Microscopic image of the synthesized clusters. (**b**) DLS analysis of clusters. (**c**) TEM image, the silica particles with an approximate size of 20 nm are surrounded by two enzymes (two dark spots with about 4 nm in size) as well as other polymer particles. (**d**) FT-IR spectrum of the upper phase before adding the nanoparticles (blue line) and after conjugation to silica nanoparticles (red line).

At the very onset of introduction, nanoparticles started to change the appearance of the solution from transparent to milky, which eventually turned in two distinct phases; the upper one with cloudy appearance and the lower one being transparent. The sample extracted from the upper phase was observed under a UV microscope. Thesuspending particles seen (Fig. 2a) were further analyzed by DLS and their size were found to be ~3.4 μm (Fig. 2b).We used TEM microscopy to finely characterize the interior structure of the particles. As seen in Fig. 2c, the silica particles with an approximate size of 20 nm are surrounded by two enzymes (two dark spots with about 4 nm in size) as well as other polymer particles. To verify whether the enzyme-silicananoconjugates were formed, we performed IR spectroscopy. The sample was IR-analyzed either with or without the nanoparticles. The comparison ofIR spectrums revealedtwo bond formations between the enzyme and the silica nanoparticles^38^,^39^ (Fig. 2d). A 12 unit shift, observed at the wave number of 3443 cm^−1^ is due to the vibration of the N-H group in Amide III band of the enzyme. It explains how the hydrogen atom (electron acceptor) on the N-H group electrostatically interacts with the oxygen atom (electron donor) of the silanol group on the surface of nanoparticles. The other shift occurring at the wave number of 1643 cm^−1^ pertains to the amide I band.

**Figure 3:**
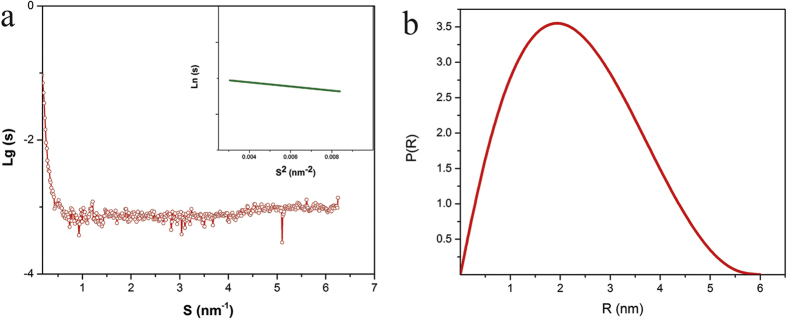
(**a**) The scattering profile of the enzyme after conjugation; The Guinier plot of the scattering profile is represented in the inset diagram. (**b**) The distance distribution function of the conjugated enzyme.

Since it was not known if the enzyme was agglomerated, we studied its structure after conjugation by SAXS analysis and interpreted the result by Guinier approximation^40^,^41^ and indirect FT-IR method, to further verify the result. The intensity scattering profile of the enzymeis obtained by subtracting the background profile of a blank solution (withno enzyme) from that of the main solution (containing enzyme). Figure 3-adepicts the intensity scattering profile of the enzyme after conjugation. According to Guinier approximation, the intensity for very small angles 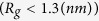 is defined as:


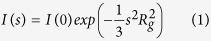


Where 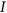
(0) is scattering intensity at zero angle, 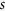 stands for scattering angle and 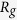 represents the radius of gyration. The slope of the linear function of *In* (1) corresponds to the radius of the enzyme. Considering a globular structure for the enzyme based on TEM observation (Fig. 1b), one can verge on the approximate radius of the enzyme by 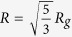 whichto 2.85 nm.

Accordant with indirect Fourier transform method^42^, the intraparticle distance distribution function is as follows:


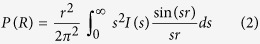


Figure 3b shows the distance distribution of the enzyme particles with an effective diameter of 2.1 nm.Indirect Fourier transform method is more accurate than Guinier approximation for it uses the entire curve of intensity scattering. There are nuances in amounts calculated and observed by DLS analysis (4 nm), suggesting the enzyme is not agglomerated.

### Aqueous two-phase system of α-amylase

The correlation proposed by Merchuk’s has been shown to be fairly capable of mathematically modeling the PEG-containingATPSs^26^,^43^. The modified expressiontogether with a mass balance equation for salt and polymer species could be written for each phase:


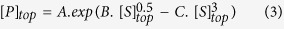



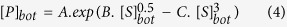


Where [*P*] and [*S*] stand for polymer and salt concentrations. *A, B and C* adjustable parameters are available for different systems beforehand^33^. Also,we can have mass balance equation as:


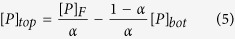



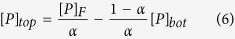


**Figure 4:**
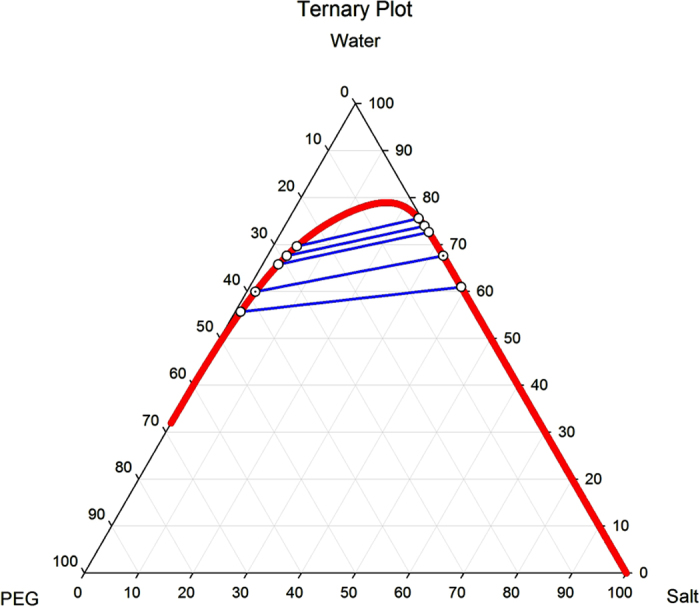
Biphasic diagram of different ATPSs; PEG6000+MgSO_4_ offers the widest extent.

By having the above equations simultaneously solved, the phase compositions will be determined. Figure 4 represents ourdata that we explored for different systems. As the ternary plot shows, PEG 6000 provides the widest bi-phasic region.

### Partitioning of α-amylase

The partition coefficient of the enzyme in ATPS is defined as:


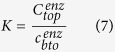


Where the enumerator and the denominator are weight fraction of the enzyme inthe upper and lower phases. Table 1 summarizes the partition coefficients of α-amylase for systems studied either in the presence or absence of the silica nanoparticles. Clearly there exist a significant difference in the partition coefficient and the yield (Table 1). A possible warrant for the enhancement in recovery is the formation of enzyme-nanosilica-PEG carriers. At pHs higher than its isoelectric point (pH = 3.5), silica is hydrated and silanol anchors cover the surface of nanoparticle. From one side, the positively charged polymer adheres to the nanoparticle and from the other side, the enzyme annexes them on the surface of silicacomprising a biomolecule carrying conjugate, which owing to PEG being the main constituent, incorporates the upper phase. High amounts of surface provided by nanoparticles enable the enzyme to attach abundantly,and thus initiating the forced delivery.

### Conformational study of the enzyme

Different conformations of an enzyme can directly affect its functionality. We observedno agglomeration taking place during the conjugation. However, the conformational structure of the conjugated enzyme remained undiscovered till we performed structural analysis on it.

**Figure 5:**
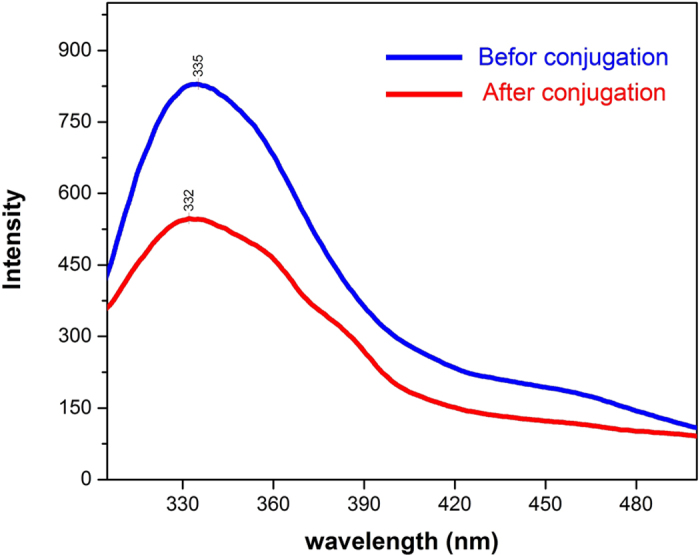
The fluorescence emission spectra of alpha-amylase before (blue line) and after conjugation (red line).

Fluorescence emitting characteristics of protein-quantum dot conjugates is prevalently used to probe the changes in native structure of an enzyme. It is due to the fluorescence of their constituent aromatic amino acid residues that enzymes exhibit such a property. Having numerous tryptophan (Trp) groups, α-amylase absorbs the light at the wavelength of 280 nm and re-emits it between 300 to 350 nm^44^. It has been observed that when an enzyme (which can act as a chromophore) binds onto an oxide nanoparticle (quencher), its fluorescence quenches^45^,^46^. That is, when studied by PL spectroscopy, the absorbance peak intensity decays. The decay is accompanied by a shift toward higher or lower wavelengths. In case of α-amylase if tryptophan residues turn inward, the emission blue-shifts. The red shift is usually observed in the event that the enzyme unfolds and tryptophan residues are exposed inthe solvent. Figure 5 represents the fluorescence emission spectra of the system, before and after conjugation. The decrease observed corroborates to the fact that the conjugation was formed and the small blue shift indicates that the enzyme was partially folded.

By intuition, it was expected that the enzyme wouldexperience folding, which is contrary to the results of PL analysis; we, therefore, did notcircumscribe our observations by PL analysis and performed detailed investigation on the structure of the enzyme: the secondary and the tertiary structures. Several methods have been developed to define the structure of proteins in solutions amongst which circular dichroism (CD) and FT-IR spectroscopy are the most versatile. Since the source of error for each method differs, one can verify the other. Owing to the ordered chiral structure of their constituent amino acids, enzymes exhibit dichroism when exposed to polarized light^47^,^48^. This intrinsic property offers an excellent clue to determine their secondary structure. Detecting differential absorption of polarized light, CD spectroscopy can rapidly determine the secondary structure and folding of a protein either when it is dissolved or adsorbed on a surface^49^,^50^,^51^.

**Figure 6:**
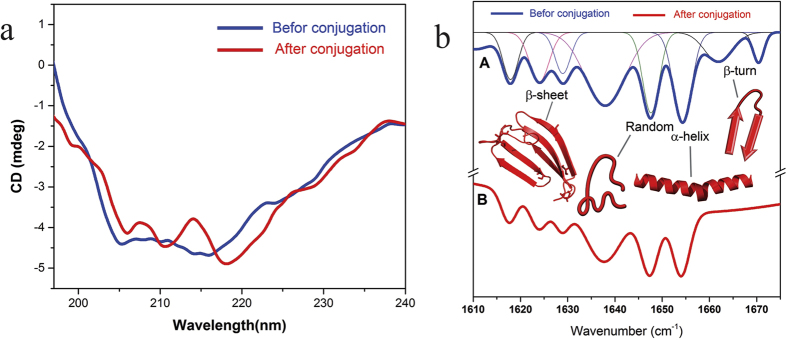
Structural studies of the enzyme. (**a**) The CD spectra of alpha-amylase enzyme before (blue line) and after (red line) conjugation. (**b**) The peak positions, their corresponding secondary structures, and the fitted Gaussian curve of the alpha-amylase.

The secondary structure of the enzyme was examined by FT- IR spectroscopy as well. As pointed out earlier, the amide I band in enzymeis very sensitive to the structural changes. Each different attracting frequency in the amide I interval (1600–1700 cm^−1^) results in aspecific secondary structure. This is due to different molecular geometry and an exclusive hydrogen bond pattern. In order to determine the secondary structure of α-amylase via IR spectroscopy, the enzyme structure was assumed to be a linear combination of the common secondary structures. The weight of each secondary structure was determined by proportion corresponding to its peak area^52^,^53^. Figure 6a represents the CD spectra of the enzyme before and after conjugation; the peak positions, their corresponding secondary structures, and the fitted Gaussian diagrams are represented in Fig. 6b. These peaks were obtained by the secondorder derivative method. Table 2 summarizes the average of FT-IR and CD results.

**Figure 7:**
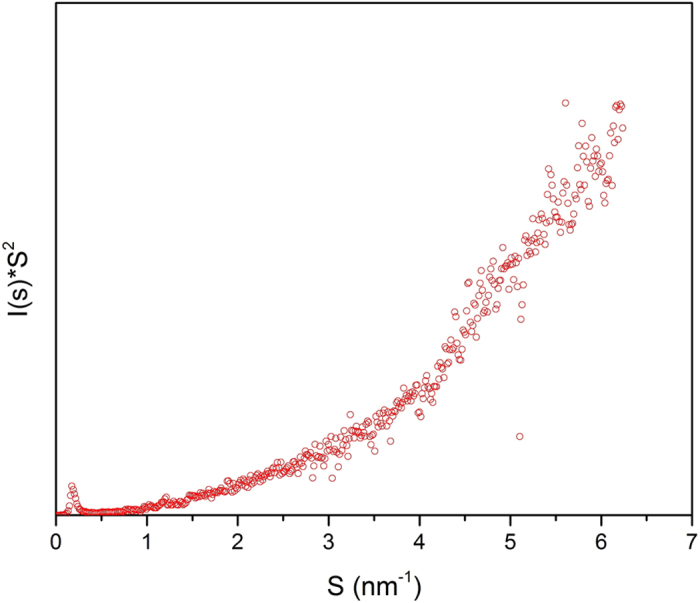
The Kratky plot of the enzyme. The observed small peak in low S and the smooth growth of I(s).S^2^ confirms that the protein is partially unfolded.

To determine the tertiary structure of the enzyme, we adopted the Kratky’s method to interpret the SAXS analysis data^54^,^55^. Figure 7 shows the Kratky plot of conjugated enzyme. Folding of globular proteins typically yield a conspicuous peak at low S; whereas, unfolded proteins show a continuous increase inS^2^•I(s) asS increases. The observed small peak in low S and the smooth growth of S^2^•I(s) confirm that the protein was partially unfolded.

**Figure 8:**
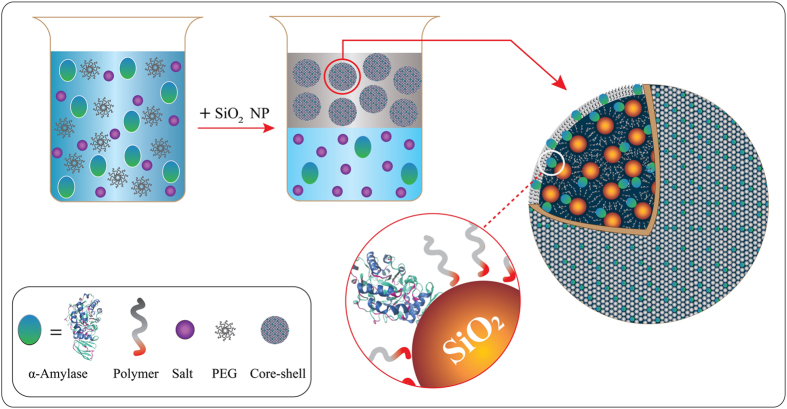
The graphical abstract, which visualizes the conjugating process: Silica nanoparticle introduced to the system acts as a bridge that connects the enzyme and polymer. These nanoconjugates, owing to the polymer being the main constituent of the upper phase, attend the upper phase and enhance the recovery.

To summarize, we enhanced the partitioning of α-amylase in aqueous two-phase system of polymer and salt by addition of silica nanoparticles. The formation of nanoconjugate enzyme carriers increases the partitioning up to seven folds. It is imperative that the enzyme retain its activity during the process of separation. We analyzed the secondary and the tertiary structure of the enzyme, which revealed the enzymewas partially unfolded. In concordance with the results of structural analysis, the DNS method showed that activity of the enzyme decreased to ~85%. Studies of similar type will helpdevelop new methods to improve bioseparation techniques and build up experimental data scarce in the field. However, there remain questions, which are open to further investigation. For example the optimal surface to volume ratio of nanoparticles for maximum adsorption of the enzyme needs to be studied.Also, the PL results, indicating enzyme to be partially folded, contradict those of SAXS analysis, which suggests that enzyme was unfolded. It would be of great importance to recognize the source of distinction. For having a quick summary on the overall process of conjugation, a concluding figure (graphical abstract) is provided in Fig. 8.

## Additional Information

**How to cite this article**: Mohsen Dehnavi, S. *et al.* PEGylated silica-enzyme nanoconjugates: a new frontier in large scale separation of a-amylase. *Sci. Rep.* **5**, 18221; doi: 10.1038/srep18221 (2015).

## Figures and Tables

**Table 1:** Phase composition of the system: Comparison of partitioning and yield before and after conjugation.

**Feed**			**Results Without N.S**			**Results with N.S**		
**% PEG**	**% Salt**	**%Water**	**α**	**K**	**Yield**	**K with N.S**	**Yield With N.S**	**%Enhancement**
18	12	70	0.629	0.791	1.167	1.471	2.173	186.1
24	18	58	0.553	0.503	0.543	1.389	1.497	275.8
12	18	70	0.38	0.43	0.61	1.88	2.67	436
18	18	64	0.47	0.45	0.35	1.37	1.04	301
24	6	70	0.6	0.388	0.506	1.324	1.728	341.6

**Table 2:** The secondary structure of α–amylase. Before and after conjugation.

**Secondary structure**	**Before conjugation (%)**	**After conjugation (%)**
α–helix	15.7	21.2
β- sheet	37.1	28.3
β- turn	16.2	0
Random coil	31	50.6
